# The role of peptides in bone healing and regeneration: a systematic review

**DOI:** 10.1186/s12916-016-0646-y

**Published:** 2016-07-11

**Authors:** Ippokratis Pountos, Michalis Panteli, Anastasios Lampropoulos, Elena Jones, Giorgio Maria Calori, Peter V. Giannoudis

**Affiliations:** Department of Trauma & Orthopaedics, School of Medicine, University of Leeds, Leeds, UK; Unit of Musculoskeletal Disease, Leeds Institute of Rheumatic and Musculoskeletal Medicine, St. James University Hospital, University of Leeds, LS9 7TF Leeds, UK; Department of Trauma & Orthopaedics, School of Medicine, ISTITUTO ORTOPEDICO GAETANO PINI, Milan, Italy; NIHR Leeds Biomedical Research Unit, Chapel Allerton Hospital, LS7 4SA Leeds, West Yorkshire, Leeds, UK

**Keywords:** Peptides, Mesenchymal stem cells, Bone healing, Growth factors

## Abstract

**Background:**

Bone tissue engineering and the research surrounding peptides has expanded significantly over the last few decades. Several peptides have been shown to support and stimulate the bone healing response and have been proposed as therapeutic vehicles for clinical use. The aim of this comprehensive review is to present the clinical and experimental studies analysing the potential role of peptides for bone healing and bone regeneration.

**Methods:**

A systematic review according to PRISMA guidelines was conducted. Articles presenting peptides capable of exerting an upregulatory effect on osteoprogenitor cells and bone healing were included in the study.

**Results:**

Based on the available literature, a significant amount of experimental in vitro and in vivo evidence exists. Several peptides were found to upregulate the bone healing response in experimental models and could act as potential candidates for future clinical applications. However, from the available peptides that reached the level of clinical trials, the presented results are limited.

**Conclusion:**

Further research is desirable to shed more light into the processes governing the osteoprogenitor cellular responses. With further advances in the field of biomimetic materials and scaffolds, new treatment modalities for bone repair will emerge.

## Background

After a traumatic insult to the bone, the musculoskeletal system mounts both local and systemic reactions facilitating the prompt restoration of the continuity of bone and normal function. Unfortunately, this process is not always successful. Approximately 5 % to 10 % of the fractures occurring are associated with impaired healing, including delayed union or non-union [1–5]. Fracture non-union often results in devastating outcomes for the patient and the surgeon [2, 5, 6], requiring a complex, long-lasting and expensive treatment, and a variable degree of morbidity is often a common finding [2, 7, 8].

In established non-unions and bone defects, bone grafting is a common procedure. It is estimated that 1.5 million bone grafting procedures are performed annually in the USA and this figure is rapidly increasing due to population ageing [2, 7, 9–13]. The intense research in this field seen over the last few decades, has resulted in the discovery of several proteins that can upregulate the bone healing response [14, 15]. Bone morphogenetic proteins (BMPs) are the most representative example, which have been granted US Food and Drug Administration (FDA) approval for clinical applications in recalcitrant long bone non-unions, lumbar fusion and open tibial shaft fractures [16–18]. Several other proteins have shown to upregulate the osteogenic bone healing process [19–22]. However, the high cost derived from the purification techniques and the high doses required due to the instability of these molecules in vivo are the two most significant points of concern [23]. Recombinant DNA technologies have simplified the production of these molecules and the discovery of a variety of osteogenic peptides has emerged [24].

The terms protein, polypeptide, oligopeptide and peptide are rather ambiguous and overlapping in their meaning [25]. Proteins usually refer to denote an entire biological molecule in a stable conformation, while peptides refer to short amino acid oligomers most commonly lacking a stable 3-dimensional structure. In general, they exert their effect through binding to specific high-affinity receptors on the respective target cell receptors [25].

To date, a number of peptides have been engineered to upregulate the osteogenic response. Although BMP-derived peptides are the most studied, other peptides also exist. The aim of this study is to identify the currently existing osteogenic peptides, other than those derived from BMPs and to investigate their impact in the upregulation of bone healing and bone regeneration.

## Methods

This review was conducted in accordance to the PRISMA guidelines [26]. Publications from January 1980 to date were included.

### Eligibility and exclusion criteria

Studies selected were original articles publishing results on the effect of different peptides on osteoblasts and osteoprogenitor cells as well as in vivo studies on bone healing. All studies that did not fulfil all eligibility criteria were excluded from further analysis. Exclusion criteria included manuscripts in languages other than English and those with incomplete documentation. Also, peptides related to BMPs or those related to cells types or conditions distant to bone healing or bone cells were excluded from the selection process as these were out of the scope of the manuscript.

### Information sources

Studies were identified by searching PubMed Medline, Ovid Medline, Embase, Scopus, Google Scholar, and the Cochrane Library to retrieve all available relevant articles. The terms used for the search included combinations of primary keywords including ‘peptide’, ‘sequence’, and ‘motif’ with secondary keywords including ‘bone’, ‘osteoblast’, ‘bone healing’, ‘mesenchymal’, ‘fracture’, ‘non-union’, ‘osteoprogenitor cells’, ‘stem cells, ‘growth factor’, and ‘extracellular matrix’. The identified articles and their bibliographies, including any relevant reviews, were manually searched for additional potential eligible studies.

### Study selection

Two of the authors (IP, MP) performed the eligibility assessment in an independent, unblinded and standardised manner. Most citations were excluded on the basis of information provided by their respective title or abstract. In any other case, the complete manuscript was obtained, scrutinised by the two reviewers and included if fulfilling the eligibility criteria.

## Results

**Fig. 1:**
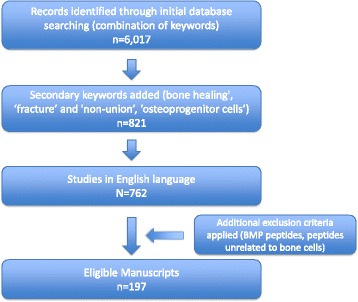
Flowchart of study selection process

Out of 6017 papers that were initially identified, 197 met the inclusion criteria (Fig. 1) [27–223]. These studies are presented below.

### Parathyroid hormone-related peptides

#### Parathyroid hormone 1–34 peptide (Teriparatide)

**Fig. 2:**
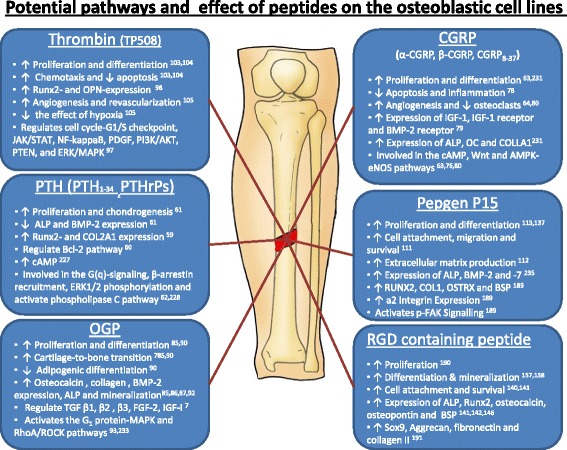
Potential pathways and effect of peptides on the osteoblastic cell lines

Parathyroid hormone (PTH) is an 84-amino acid, naturally occurring protein that plays a major regulatory role in mammalian mineral ion homeostasis. The peptide derived from its 34 amino acid domain has similar activity to the full length protein [224]. PTH_1–34_ is one of the earliest artificially synthesized amino acid fragments that was granted approval for the prevention and treatment of osteoporosis. Among its several functions, PTH_1–34_ stimulates osteoblast proliferation, differentiation and prevents their apoptosis (Fig. 2) [51].

Synthetic matrix made of polyethylene-glycol containing PTH_1–34_ significantly stimulated in situ bone augmentation in rabbits [29]. Evidence from animal models shows that daily subcutaneous injections of PTH_1–34_ significantly increased the bone mineral content and density as well as the total osseous tissue volume, torsional strength and stiffness [27, 30]. Additionally, accelerated callus mineralization, increased bone density at the fracture site, and better mechanical properties of the united bone have been reported [27, 31, 32, 47–51].

To date, several case reports have indicated that teriparatide could facilitate the healing of sternal non-union [34], stress fractures [35], atrophic humeral shaft non-union [36], femoral non-union [37, 41, 42, 47, 225], hip fractures [40], delayed unions [38, 43, 44], periprosthetic fractures [45], and sacral and pubic insufficiency fractures [39].

In a prospective randomized double-blind study, Aspenberg et al. [28] analysed the effect of daily injections of 20 and 40 μg of recombinant teriparatide against placebo in post-menopausal women who sustained a distal radial fracture. Although they reported no significant difference between the teriparatide 40 μg versus placebo groups, they reported a shorter time to healing between the teriparatide 20 μg group and placebo (*P* = 0.006). In a post hoc subgroup analysis by the same group, blinded qualitative scoring of the calluses at 5 weeks suggested that patients who received PTH_1–34_ had a more ‘rich’ callus formation [33].

#### Parathyroid hormone-related protein

Human parathyroid hormone-related protein (PTHrP) consists of 139–175 amino acids and is a key regulator of cell growth, differentiation, and development of the foetal skeleton [59–62, 226–228]. Endogenous PTHrP plays an important role in fracture healing as demonstrated in an PTHrP haplo-insufficiency model where reduced cartilaginous and bony callus formation was noted together with reduced endochondral and osteoblastic bone formation [229]. However, a PTHrP_1–34_ maintains a less pronounced anabolic effect to the bone and osteoblasts possibly due its higher clearance rate [52, 53]. To overcome this weakness, several analogues have been developed to date [54–57].

PTHrP_1–36_ exerts an anabolic action to bone, including enhanced bone histological features and raised osteoblast differentiation markers in the long bones and plasma in mice [57]. Cavitary bone defects treated with PTHrP_107–111_ improve local bone induction in a rabbit femoral cavity defect model [58]. A C-terminally substituted analogue of PTHrP_1–34,_ the RS-66271, was found to increase trabecular and cortical bone in ovarectomized osteopenic rats [54]. In an impaired bone healing animal model, daily injections of RS-66271 resulted in a larger callus area, greater stiffness and torque when compared with controls [56]. A similar analogue of PTHrP, the RS-50303, was found to enhance fracture healing in a rat femoral osteotomy model [55].

### Calcitonin gene-related peptide

Calcitonin gene-related peptides (CGRP) are found in two forms, α and β. α-CGRP derives from the *Calca* gene and consists of 37 amino acids [230]. It has a 20 % homology with calcitonin. In contrast, β-CGRP derives from a separate gene, termed *Calcb*, which is located in close proximity to *Calca.* In bone, CGRP is found in the sensory nerve endings in periosteum, bone marrow and metaphysis [66]. Among its several functions, CGRP has been found to stimulate the proliferation and differentiation, and to reduce the apoptosis of osteoprogenitor cells [63, 64, 75, 79, 231]. CGRP levels increase in patients with fractures, and it has been postulated that this plays an important role during the inflammatory stage of bone healing and overall during damaged tissue repair [72, 80]. Transgenic mice engineered to overexpress CGRP have been found to have high trabecular bone density and bone volume [65], also associated with an increase in bone formation rate. On the contrary, α-CGRP null mice developed osteopenia caused by a reduced bone formation rate [69]. CGRP was also found to stimulate the production of osteogenic molecules like IGF-I and BMP-2 [76–78].

Despite the abovementioned experimental evidence, limited in vivo studies have explored the potential supplementary effect of CGRP on bone healing. The literature suggests that, during fracture healing, the systemic levels of CGRP increase [72]. Fracture evokes an intense local in-growth of new nerve fibres containing CGRP thus possibly playing a role in the bone healing process [71]. When the bony innervation is disrupted locally, this results in an insufficient fracture callus [73]. Furthermore, Song et al. [70] speculated that the increased levels of CGRP secondary to traumatic brain injury may have led to the enhancement of fracture healing seen in this patient group. In an experimental model of induced fatigue bone damage, administration of CGRP or CGRP(8–37) increased reparative bone formation [74].

### Osteogenic growth peptide (OGP)

OGP is a naturally occurring, highly conserved, 14-amino acid, H4 histone-related peptide [81], abundant in human and mammalian blood as well as in culture media of osteoblasts and fibroblasts [81, 82]. Following its dissociation from the a_2_-macroglobulin, the peptide is proteolytically cleaved to generate a C-terminal pentapeptide, which activates an intracellular Gi-protein-MAP kinase signalling pathway [232, 233].

OGP was found to exert an anabolic effect on bone cells, resulting in an increase of bone formation and overall bone mass [81, 83]. In vitro studies have shown that OGP can regulate osteoprogenitor cell proliferation, differentiation, alkaline phosphatase activity, osteocalcin secretion, collagen and matrix mineralization [85–87, 92]. In vivo OGP was found to regulate TGF-b1,b2,b3, FGF-2, IGF-I and aggrecan [85]. Further, transgenic mice overexpressing OGP have significantly increased peak bony mass [91].

Experimental fracture healing models have shown that OGP can serve as a potential candidate in enhancing the bone healing response (Table 1) [83–85, 88, 89]. Systemic administration of OGP accelerated bony union with enhanced bridging across the fracture gap, higher volume of callus and newly formed bone [85, 89]. Shuqiang et al. [84] treated 1.5-cm segmental defects in rabbits with an OGP incorporated in a PLGA scaffold. Their results showed a higher bony volume and acceleration of bone healing response.

**Table 1:** In vivo animal studies presenting the effect of osteogenic growth peptide (OGP) on bone and bone healing

Study/Year	Model	Mode of delivery	Results
Sun et al., 1998 [89]	Tibial fracture in rabbits	IV administration	• OGP treatment accelerated fracture union
Brager et al., 2000 [85]	Femoral fracture in rats	Systematic administration of OGP (25 ng/day)	• OGP enhances proliferation and differentiation of osteogenic cells possibly through the upregulation of TGF-beta
Gabet et al., 2004 [83]	Mid-femoral fracture in rats	Systematic administration of OGP	• OGP administration resulted in enhanced bridging across fracture gap, higher volume of callus and newly formed bone
Shuqiang et al., 2008 [84]	Radial 1.5 cm segmental defect in rabbits	OGP incorporated in PLGA	• The rate of bone formation and volume were statistically significantly upregulated in experimental group
Zhao et al., 2011 [88]	Distraction osteogenesis in rabbit tibia	Systematic administration of OGP (200 ng/kg/day)	• OGP treatments resulted in greater torsional stiffness, higher chondrocyte numbers and amount of newly formed bone

### Thrombin Peptide 508 (Chrysalin)

Thrombin peptide 508 (TP508), also known as Chrysalin, is a 23-amino acid synthetic peptide that represents the non-proteolytic receptor binding domain of thrombin. TP508 mimics some specific attributes of the thrombin, without the undesirable blood clotting effects. TP508 was found to enhance the proliferation and differentiation of and induces chemotaxis in human osteoblasts [103, 104]. It enhances VEGF-stimulated angiogenesis and attenuates effects of chronic hypoxia [105].

A number of in vivo animal models have all demonstrated that TP508 could have an upregulatory effect on bone healing (Table 2). Two animal studies, analysing the effect of TP508 loaded on PPF composite and microsphere scaffolds on segmental bone defects in rabbits, showed enhanced bone formation with a higher torsional stiffness of bone [99, 101]. TP508 injected into the fracture gap promotes fracture healing and increased blood vessel formation [95, 97, 102]. In animal models of distraction osteogenesis, injection of TP508 into the fracture gap resulted in enhanced bone formation and consolidation [94, 100]. In similar models, increased numbers of osteoblasts were apparent as well as the increased quality of bone [96, 98, 100].

**Table 2:** In vivo animal studies presenting the effect of TP508 on bone and bone healing

Study/Year	Model	Mode of delivery	Results
Hedberg et al., 2004 [101]	Segmental bone defect in rabbits	PPF composite scaffolds with 200 or 100 μg TP508	• Enhance bone formation with 200 μg TP508 possibly due to the initial high burst of the molecule
Sheller et al., 2004 [99]	Segmental bone defect in rabbits	Microspheres with 100 μg or 200 μg TP508	• Enhanced healing of the defects with higher torsional stiffness in the animals treated with TP508
Li et al., 2005 [100]	Distraction osteogenesis in rabbits	30 μg or 300 μg into the distraction gap	• Enhanced bone formation and consolidation, the 300 μg treatment group had the most advanced results
Wang et al., 2005 [102]	Femoral fracture in rats	1 μg or 10 μg in the fracture gap	• TP508 found to promote fracture healing by inducing the levels of growth factors, inflammatory mediators and angiogenesis-related genes
Amir et al., 2007 [98]	Distraction osteogenesis in rabbits	30 or 300 μg into the distraction gap	• Enhance bone regeneration with increased number of osteoblasts
Li et al., 2007 [97]	Rat femoral fracture model	1 μg, 10 μg or 100 μg in the fracture gap	• TP508 accelerated fracture healing by upregulating the expression levels of molecules involved in cellular proliferation, cellular growth and apoptosis
Wang et al., 2008 [96]	Distraction osteogenesis in rabbits	Slow releasing TP508 preparation (300 μg in PPF/PLGA microparticles)	• Enhanced bone consolidation process with better quality bone
Hanratty et al., 2009 [95]	High energy femoral fracture in mice	10 μg or 100 μg at fracture site, or 100 μg at muscles adjacent the fracture	• 100 μg in fracture gap significantly increased bone formation and fracture stiffness • Less scar tissue and increased blood vessel formation was noted when TP508 was injected in the adjacent to the fracture muscles
Cakarer et al., 2010 [94]	Distraction osteogenesis in rats	10 μg and 100 μg percutaneously	• Significant larger area of consolidation in the animal receiving TP508; the higher dose was more effective

In the clinical setting, TP508 has failed to display the same beneficial effects as in animal studies. A double-blinded, randomized, placebo controlled Phase III clinical trial has been conducted to analyse the effect of Chrysalin for the treatment of unstable displaced distal radial fractures [106]. The initial results demonstrated a statistically significant shorter time to the radiologic consolidation of the fractures but not differences in terms of the range of motion, grip strength and VAS or DASH scores [107]. Furthermore, the trial failed to show any statistically significant difference in the time of removal of the immobilization device, which was the primary end point of the study [108].

### Cell-binding peptides

#### PepGen P-15

The P-15 peptide is a highly conserved peptide that consists of 15 amino acids identical to the cell-binding region of collagen type I [234]. P-15 enhances cell attachment to bone substitutes and upregulates extracellular matrix (ECM) production [112]. At the same time, it promotes cell survival and can be absorbed into a calcium phosphate substrate [111]. When P-15 is added in scaffold material, it results in a significantly higher gene expression of alkaline phosphatase (ALP), BMP-2 and BMP-7 [235]. This upregulated gene expression could suggest that P-15 promotes osteoblastic activity in human osteoblast cells. Indeed, P-15 was found to stimulate the proliferation and differentiation rate as well as the growth factor production of osteoblasts in vitro [113, 137, 189]. On the contrary, Vordemvenne et al. [104] reported that P-15 alone is not capable of upregulating the proliferation and calcifying potential of human osteoblasts in vitro. When combined with PDGF, a statistically significant increase in both proliferation and calcification was noted [104].

Preclinical results have shown that P-15-containing bone graft substitutes could facilitate bone healing and regeneration [118]. In bone defects, application of P-15-containing bone substitutes increased the rate of bone growth compared to the defects left empty or filled with bone substitute alone [120, 121, 123, 124, 132, 138]. In critical sized segmental defect in a rat radius, application of inorganic bone matrix together with P-15 resulted in positive effect on bone healing, without any immunogenic features and disease transmission risk [133]. The use of the same graft material was found as successful as autogenous bone graft in producing lumbar spinal fusion in an ovine model [119]. However, some controversial data exist, with some authors reporting less favourable results with P-15-containing graft substitutes [125, 126]. In addition, application of the P-15-containing graft substitutes was found to accelerate the process of early bone formation response but not the long-term effect [129, 131, 135].

The majority of clinical evidence derives from substitutes for the oral cavity [109, 110, 114–117, 122, 127, 128, 130, 134, 136, 139]. Periodontal osseous defects in 25 patients treated with combination of anorganic bovine-derived hydroxyapatite matrix and P-15 showed favourable clinical results [109]. In the treatment of non-unions limited evidence exists [236]. Gomar et al. [236] treated 22 patients with non-uniting fractures with P-15 containing bone graft substitutes. They reported a 90 % success rate and concluded that it could be an effective, safe and economical alternative to autologous bone grafting.

#### RGD containing peptide

Arginyl-glycyl-aspartic acid (RGD) sequence is found in several molecules and constitutes a system of cell surface signalling [237]. Evidence suggests that RGD enhances cell attachment and spreading of osteoblasts onto scaffolds and graft material [140, 141, 159, 188] whilst increasing cellular proliferation and the expression of ALP, Runx2, osteocalcin, osteopontin and bone sialoprotein [141, 142, 146, 190, 191]. Further, it promotes osteoblast differentiation and mineralization [143, 144, 146, 157, 158, 197].

Limited in vivo studies exist today analysing the effect of RGD on bone healing; however, several authors have investigated the effect of RGD peptides on implant surfaces. RGD coated implants were found to have an increased peri-implant bone formation and enhanced direct bone apposition even in areas of poor surrounding bone [148, 149, 151, 152, 155, 156]. This significantly increased the bone-to-implant contact [149]. When RGD-coated intramedullary nails were inserted into the tibia of male adult Wistar rats, the outcome was increased new bone formation [148]. Finally, it should be mentioned that RGD-containing scaffolds used to deliver growth factors, such as BMP-2 to promote bone regeneration in experimental fracture models, exist with favourable results [150]. In contrast to the abovementioned results, some fracture models have shown that RGD utilization could have detrimental effects. Hennessy et al. [153] showed that, when RGD was combined with adsorbed tibial proteins like fibronectin, vitronectin and fibrinogen, a markedly detrimental effect on mesenchymal stem cell (MSC) adhesion and survival was observed. No significant effects of an additional RGD coating on HA surfaces were detected in a rabbit model for cementless joint prostheses [154].

#### Other ECM-derived peptides

In addition to P-15 and RGD, other ECM-derived peptides are currently being developed for potential applications in amplifying the bone healing response. They represent signalling domains found along the ECM protein chains and are capable of interacting with receptors on the cellular membrane.

GFOGER (glycine-phenylalanine-hydroxyproline-glycine-glutamate-arginine) is a collagen-mimetic peptide. It selectively promotes α_2_β_1_ integrin binding, which is a crucial event for osteoblastic differentiation [162]. Implants coated with GFOGER were found to improve peri-implant bone regeneration and osseointegration [162, 164]. Results showed significantly accelerated and increased bone formation in non-healing femoral defects compared to uncoated scaffolds and empty defects. GFOGER could be utilized as a growth factor delivery vehicle, which can upregulate the fracture healing response [161].

The collagen-binding motif (CBM) is a cleavage product of osteopontin that can specifically bind to collagen [180]. The CBM was found to promote migration and osteogenic differentiation via the Ca^2+^/CaMKII/ERK/AP-1 signalling pathway [181]. In a rabbit calvarial defect model, application of an injectable gel containing synthetic CBM peptide resulted in increased cell adhesion and growth of osteoblasts followed by increased osteoblastic differentiation and marked bone formation [180, 238].

DGEA (Asp-Gly-Glu-Ala) is a recognition motif used by type I collagen to bind to α_2_β_1_ integrin [165]. This collagen peptide sequence has been shown to promote cell adhesion, spreading and osteogenic differentiation [163, 165, 166]. DGEA, engineered to express a heptaglutamate domain, was found to accumulate within bone tissue following intravenous injection [168], suggesting that such an approach could be used for drug to bone delivery. DGEA coupled with heptaglutamate-containing hydroxyapatite was found to enhance the adhesion and osteoblastic differentiation of MSCs as well as to increase new bone formation and bone-to-implant contact [167].

The SVVYGLR (Ser-Val-Val-Tyr-Gly-Leu-Arg) peptide sequence is found adjacent to the RGD sequence in osteopontin [239]. SVVYGLR peptide significantly enhanced the adhesion and proliferation of MSCs but also endothelial cell activity, resulting in an upregulation of neovascularization [169, 239, 240]. Experimental models of bone defects have shown that, when SVVYGLR was implanted together with a collagen sponge, an upregulation of osteogenesis and angiogenesis was observed [169, 239].

KRSR (lysine-arginine-serine-arginine) is a heparin-binding site found in fibronectin, vitronectin, bone sialoprotein, thrombospondin, and osteopontin [173]. KRSR increased osteoblast adhesion and osteogenic gene expression [171, 172, 177, 178]. Anodized nanotubular titanium coated with KRSR, RGDS (arginine-glycine-aspartic acid-serine) and molecular plasma deposition increased osteoblast density compared with uncoated substrates [174]. Likewise, KRSR and RGD coated on titanium promoted the greatest osteoblast densities relative to untreated titanium [175]. On the contrary, less favourable results in terms of stimulation of cell adhesion and spreading were reported by other studies [173, 179].

FHRRIKA (Phe-His-Arg-Arg-Ile-Lys-Ala) is a cell-binding and putative heparin-binding domain of bone sialoprotein. FHRRIKA could have a favourable effect on osteoblast adhesion, spreading and mineralization [183]. Osteoblast outgrowths from rat calvarial bone chips covered a significantly larger area on FHRRIKA surface [176]. Rat calvarial osteoblasts seeded into a scaffold containing the RGD and FHRRIKA sequences were found to remain viable and have higher proliferation kinetics compared to the controls in which no peptides were added [184].

Fibronectin (FN)-derived peptides have also shown to facilitate osteoblast adhesion, spreading and mineralization [185]. A fibrin-binding synthetic oligopeptide derived from FN was found to enhance new bone formation in rabbit calvarial defect model [187]. In addition, the multifunctional FN III9-10/12-14 greatly enhanced the regenerative effects of BMP-2 and PDGF-BB in a rat critical-size bone defect model [186].

### NEMO-binding domain peptide (NBD)

The inhibitor of nuclear factor kappa-B kinase (IKK) is a high molecular weight complex consisting of two catalytic subunits (IKK-1 and IKK-2) and a non-catalytic regulatory subunit NF-kB Essential Modulator (NEMO or IKK-γ) [194]. NEMO interacts with both IKK subunits at the interacting region to amino acids 737–742, called the NEMO-binding domain (NBD) [194]. NBD peptide has shown to promote osteoblast differentiation and inhibit bone resorption [192, 193]. A protective role to the bone by blocking osteoclastogenesis and bone erosion in inflammatory arthritis was also noted [195]. In vivo evidence is limited to a murine tooth extraction model treated with lipopolysaccharide injection where TNF-a retarded bone regeneration [196].

### Cell penetrating peptides

Cell penetrating peptides (CPPs) are peptides that can transverse the cellular membrane and transport their ‘cargo’ into the cytoplasm [241]. Such cargos include proteins, siRNA, nanoparticles, oligonucleotides, and other peptides [242]. CPPs can derive from bacteria and viruses or synthesized in the laboratory [241, 242]. Jo et al. [199] demonstrated that the CPP-conjugated co-activator-associated arginine methyltransferase 1 (CARM1) protein can be delivered into human MSCs and change their global gene expression profile. Furthermore, upregulation of their differentiation capacity was noted [199]. In a rabbit calvarial defect model treated with CPP with a transcriptional factor fusion protein resulted in significantly increased bone formation [200]. Similarly, in a critical-size calvarial defect model, the inclusion of tetrameric CPPs in ex vivo transduction of recombinant adenovirus expressing BMP-2 into MSCs promoted highly mineralized bone formation [201].

### Self-assembly peptides

Self-assembly peptides are another class of peptides, referred by some as ‘molecular Lego’, that are composed of alternating hydrophilic and hydrophobic amino acid residues [243]. These residues have the tendency to spontaneously adopt a β-sheet structure when exposed to monovalent cation solutions or placed under physiological conditions [203, 243]. The outcome of this process is the formation of self-assembled matrices with interwoven nanofibers.

RADA16-I (AcN-RADARADARADARADA-CONH2) is a synthetic commercially available peptide (PuraMatrix). MSCs exhibited higher levels of expression of ALP, osteocalcin and Runx2 genes in RADA16-I-containing demineralized bone matrix (DBM) compared to only DBM [203]. Cell adhesion, proliferation and differentiation of osteoblasts were found to be superior in the RADA16-I-containing scaffold [204]. In vivo data derived from a critically-sized femur defect in goats showed that the volume of newly formed bone from marrow-enriched RADA16-I/DBM was significantly higher compared to marrow-enriched DBM alone [203]. Other authors reported favourable outcomes with the utilization of RADA16-I self-assembly peptide [206–212]. The addition of BMP-2 in a hydrogel RADA16-I-containing scaffold significantly enhanced bone regeneration on the bone augmentation model in an animal bone defect model [205].

Peptide amphiphiles are another class of self-assembly peptides that can support osteoprogenitor cells and guide their differentiation [215, 216]. Mineralized matrices containing peptide amphiphiles were found to promote osteogenic differentiation of human MSCs [213]. The combination of peptide amphiphiles with MSCs and platelet-rich plasma was found to promote bone formation and enhance angiogenesis [214].

### Other peptides

Numerous peptides have been isolated from the majority of the existing growth factors and bone-related proteins. Peptides derived from fibroblast growth factor were found to upregulate osteoblast differentiation [202, 244]. Similarly, peptides have been derived from molecules like BMPs, transforming growth factor-β, vascular endothelial growth factor, insulin derived growth factor, although their potential role in bone healing and regeneration remains obscure [217–219, 222]. Other peptides found to promote bone healing include the RANKL-binding peptide, AC-100, mechano growth factor E, and B2A2-K-NS (B2A) [223].

## Discussion

Bone tissue engineering is a growing biomedical field. All recent advances in the field of growth factors, scaffolds and osteoprogenitor cells have boosted the application and further expansion of tissue engineering technologies. As far as growth factors are concerned, several drawbacks prohibit their widespread use. Difficulties arising from potential immunogenicity, large molecular weight, need for carriers for their delivery and instability in vivo are well recognized [188]. Moreover, concerns regarding their sterilization and their theoretical involvement in carcinogenesis also exist [23, 188, 245–247]. The discovery that small protein segments (peptides) have the capacity to exert a similar effect could overcome some of the abovementioned problems. Not only do they have low immunogenicity but they can also be easily synthesised and handled [188].

Chrysalin and teriparatide are two commercially available drugs that have been investigated as potential candidates in the upregulation of bone healing response in humans [28]. Their background in terms of pre-clinical and experimental evidence has been excellent. In humans, teriparatide resulted in a shorter time to healing with a ‘richer’ callus formation when used for the treatment of distal radial fractures [28]. These results, however, are rather weak and, according to the authors, they should be interpreted with caution and warrant further validation with more studies. Similarly, the use of Chrysalin for unstable displaced distal radial fractures demonstrated a shorter time to the radiologic consolidation of the fractures but no differences in terms of cast removal, range of motion, grip strength and VAS or DASH scores [107]. Therefore, one could hypothesise that the ‘exceptional’ results seen in the experimental animal models cannot be directly translated in clinical practice, at least as yet. It could be speculated that the differences in bone healing biology are responsible for these compelling results. In addition, differences in the study objectives in humans and animals are evident. The available in vitro and in vivo animal studies have limited their focus on the global osteogenic output. However, clinical studies are not limited to the radiologic appearance, but also several other parameters such as time for cast removal, range of motion, VAS or DASH scores, etc. It could be of speculation that a more radiologically rich callus formation is not necessarily associated with a better clinical outcome (Table 3). In addition, the potency of these peptides in humans and animals, as well as their stability and delivery challenges, are currently not fully understood.

**Table 3:** Available clinical studies on the effect of peptides on bone healing

Study, Year	Peptide used	Clinical application	Result
Yukna et al., 1998 [115]	P-15	Periodontal osseous defects in 33 patients	• P-15 combined with anorganic bone matrix (ABM) yields better clinical results than freeze-dried bone allograft or open flap debridement
Yukna et al., 2000 [114]	P-15	Periodontal osseous defects in 33 patients	• P-15 combined with ABM yields better clinical results than the ABM alone
Yukna et al., 2002 [122]	P-15	Infra-bony periodontal defects in 25 patients	• Favourable 3-year results with P-15 combined with ABM suggest that it may have a beneficial effect long-term
Yukna et al., 2002 [109]	P-15	Periodontal regeneration case report	• Uneventful results with no evidence of root resorption, ankylosis or untoward inflammation
Degidi et al., 2004 [128]	P-15	Maxillary sinus augmentation in 7 patients	• Bone-replacement materials, without the addition of autologous bone, could be equally effective sinus augmentation
Gelbart et al., 2005 [110]	P-15	Sinus floor augmentation in 12 patients	• New trabecular bone is formed after grafting P-15 combined with ABM in the sinus floor
Philippart et al., 2005 [130]	P-15	Maxillary sinus floor grafting performed on 3 patients	• High degree of inorganic xenograft integration and natural bone regeneration
Gomar et al. 2007 [236]	P-15	Non-unions and delayed union in 22 patients	• Full consolidation was achieved in 90 % of the cases • Safe, economical and clinically useful alternative to autograft in the repair of un-united fractures
Kasaj et al., 2008 [127]	P-15	Infra-bony periodontal defects in 26 patients	• Significantly improved clinical outcomes compared to open flap debridement
Butz et al., 2011 [116]	P-15	Sinus floor augmentation in 24 patients	• All implants placed in the augmented sites integrated and were restored prosthetically
Emam et al., 2011 [117]	P-15	Sinus floor augmentation in 24 patients	• PepGen P-15 putty was found to be a promising osteoconductive graft for sinus augmentation, supporting immediate placement of implants
Aspenberg et al., 2010 [33]	Teriparatide	Distal radial fractures in 27 patients	• The results must be interpreted with caution • Radiographic quality at an early time point might be a sensitive variable, perhaps better than time to cortical continuity • Teriparatide appeared to improve early callus formation in distal radial fractures
Aspenberg et al., 2010 [28]	Teriparatide	Distal radial fractures in 102 patients	• Shortened time to healing for teriparatide group compared with placebo • These results should be interpreted with caution and warrant further study
Chintamaneni et al., 2010 [34]	Teriparatide	Sternal fracture non-union	• Consolidation of fracture
Oteo-Alvaro et al., 2010 [36]	Teriparatide	Humeral shaft non-union case report	• Consolidation of fracture
Chrysalin trial [106]	Chrysalin	Distal radial fractures in 274 patients	• Statistically significant shorter time to the radiologic consolidation of the fractures but no differences in terms of the range of motion, grip strength and VAS or DASH scores

PepGen P-15 is another commercially available peptide that has been investigated in periodontal osseous defect models with favourable results. The vast majority of the available evidence comes from small osseous defects seen in dental and maxillofacial surgery (Table 3). There is limited evidence for long bone bony defects or non-unions. In the largest case series, PepGen P-15 containing bone graft substitutes were used in 22 patients with non-uniting fractures [236]. According to the authors, PepGen appeared to offer a safe, economical and clinically useful alternative to autologous grafting. However, additional randomized clinical studies are needed to define its effectiveness in this setting. In a similar note, the effectiveness of PuraMatrix warrants further clinical investigation as, although commercially available, its potential effectiveness for bone healing and regeneration is only limited to in vitro or animal studies.

One avenue that warrants further investigation includes the combination of cell binding peptides with sub-functional doses of BMPs [197, 248]. As shown, for instance, by Visser et al. [248], when an absorbable collagen type I sponge functionalized with a synthetic collagen-targeted RGD containing low doses of BMP-2, ectopic bone formation was observed in rats. These low BMP-2 levels would have no significant effect if applied on their own.

Further research in the nanoscale phenomena governing biological materials and the heterojunction between cells and substrate could allow osteoinductive implants coupled with osteoconductive properties. Small molecules such as peptides could have a role to play in supporting and guiding the overall osteogenic response in such scenarios. Overcoming the peptide stability issues against proteolysis, which result in a short duration of activity and low bioavailability, is also crucial. In this context, expansion of our methodology for peptide designs with further research on ways to improve the incorporation of non-natural amino acids, cyclization and stable peptide bond engineering are crucial. The development of improved peptide motifs that could increase the osteogenic response in a compromised bone healing environment rather than cause an upregulation of the osteoblastic output, should further be explored. Another area of interest is the utilization of a ‘polytherapy’, i.e. the combination of several peptides targeting either a specific cell line or a specific phase of bone healing. Such an approach would, for instance, employ an osteoinductive peptide coupled by a peptide promoting the osteogenic or chondrogenic response. Scaffold technologies enabling a timed controlled release of such molecules could provide the right signals at the exact phase of the bone healing pathway. Therefore, further persistence in the design of peptide-scale molecules capable of targeting the upregulation of osteogenesis or form functional, structurally complex and well-defined scaffolds will lead to future clinical treatment modalities ranging from tissue replacement to tissue regeneration.

## Conclusion

A significant number of peptides have been developed and investigated as potential candidates for the upregulation of bone healing response. In vitro and experimental animal models have been favourable, however, limited clinical evidence exists. Maturation of our knowledge in this field will give rise to novel biologically-derived molecules for applications in the clinical setting in cases where bone healing and bone regeneration are needed.
